# 10 Years of Toxicogenomics section in Frontiers in Genetics: Past discoveries and Future Perspectives

**DOI:** 10.3389/fgene.2022.979761

**Published:** 2022-09-12

**Authors:** Douglas M. Ruden

**Affiliations:** Institute of Environmental Health Sciences, C. S. Mott Center for Human Health and Development, Department of Obstetrics and Gynecology, Wayne State University, Detroit, MI, United States

**Keywords:** toxicology, toxicogenomics, epigenomics, environmental health, genomics, transcriptomics

## Abstract

The Frontiers Media family has over 200 journals, which are each headed by usually one Field Chief Editor, and several specialty sections, which are each headed by one or more Specialty Chief Editors. The year 2021 was the 10th anniversary of the founding of the Frontiers in Genetics journal and the Frontiers in Toxicogenomics specialty section of this journal. In 2021, we also announce one of the newest of the Frontiers journals–Frontiers in Toxicology which is part of the Frontiers Media family of journals but independent of Frontiers in Genetics. The Specialty Chief Editor of Toxicogenomics, and one of 26 Specialty Chief Editors of Frontiers in Genetics, is Dr. Ruden. As of 2021, Toxicogenomics has published over 138 articles and has over 370 Editors including 90 Associate Editors and 280 Review Editors. The Frontiers in Genetics impact factor was initially approximately 2.5 when it was first listed in PubMed in 2015 and has risen steadily to its current value of 4.8, which is typical for the majority of the over 200 Frontiers journals that have established impact factors. In this overview of the first decade of Toxicogenomics section, we discuss the top 5 articles with the highest Scopus citations, which were all written in the first few years of the journal. The article with the highest number of citations, with 353 Scopus over 600 Google Scholar citations, and the highest average number of citations (67) that steadily increased from 10 citations in 2013 to 119 citations in 2021, was written in 2012 by Dr. Ruden’s laboratory and titled, “Using *Drosophila melanogaster* as a model for genotoxic chemical mutational studies with a new program, SnpSift.” The five most influential authors who published in the journal in the past 10 years based on Scopus citations of a particular paper are Dr. Ruden’s laboratory, with 353 Scopus citations for the SnpSift paper mentioned above; Drs. Brock Christensen and Carmen J. Marsit, with 86 Scopus citations for their review, “Epigenomics in environmental health”; Dr. Michael Aschner and colleagues, with 61 Scopus citations for their paper “Genetic factors and manganese-induced neurotoxicity”; and Dr. Sandra C. dos Santos and colleagues, with 59 Scopus citations for their paper, “Yeast toxicogenomics: genome-wide responses to chemical stresses with impact in environmental health, pharmacology, and biotechnology.” While the top 5 papers were published in the early years of the journal, we will also discuss a more recent article published in 2018 on a comparison of RNA-seq and microarray methods by Dr. Michael Liguori’s laboratory, “Comparison of RNA-Seq and Microarray Gene Expression Platforms for the Toxicogenomic Evaluation of Liver From Short-Term Rat Toxicity Studies”, that far exceeds the number of downloads and views of all the other articles published in the first 10 years of the journal and will likely be a top cited paper in the second decade highlights of this journal. Finally, we discuss where the Toxicogenomics specialty section will go to advance the field of toxicogenomics, and more generally, toxicology, in the future.

## 1 Introduction

Frontiers Media is a publisher of peer-reviewed open access scientific journals that was founded in 2007 by a group of neuroscientists. Accordingly, the first journal published was Frontiers in Neuroscience, which opened for submission in 2007. In 2010 and 2011, Frontiers launched a series of other journals in medicine and science. In 2011, the year Frontiers in Genetics was founded, Dr. David Allison, the founding Editor in Chief of Frontiers in Genetics, asked me if I wanted to be the Specialty Chief Editor on a Toxicology specialty journal for the Frontiers in Genetics. Honored, I agreed and suggested the name Toxicogenomics because toxicogenomics was my primary research at the time–namely, studying genetical genomics of lead exposure in *Drosophila melanogaster* (Ruden et al., 2009; Ruden, 2011). In February 2013, the Nature Publishing Group (NPG) (now Nature Research) acquired a controlling interest in Frontiers Media. Last year, in 2020, the Associate Editors, Editorial Managers, and decided to start a new Frontiers Media journal, Frontiers in Toxicology, to expand the type of toxicology articles that are submitted. Currently, my laboratory, while still focused on toxicology studies, moved from toxicogenomics analyses to more transcriptomic level and epigenomics analyses (Senut et al., 2012; Senut et al., 2014; Sen et al., 2015a; Sen et al., 2015b; Sen et al., 2015c; Qu et al., 2017a; Qu et al., 2017b; Ruden et al., 2017; Sen et al., 2017; Qu et al., 2018; Shah et al., 2019; Shah et al., 2020). In this article, I give a short overview of past discoveries, current challenges, and future perspectives for the revitalization of the Toxicogenomics section.

## 2 Overview: 10 years of Toxicogenomics specialty section in Frontiers in Genetics

### 2.1 Past discoveries

**FIGURE 1:**
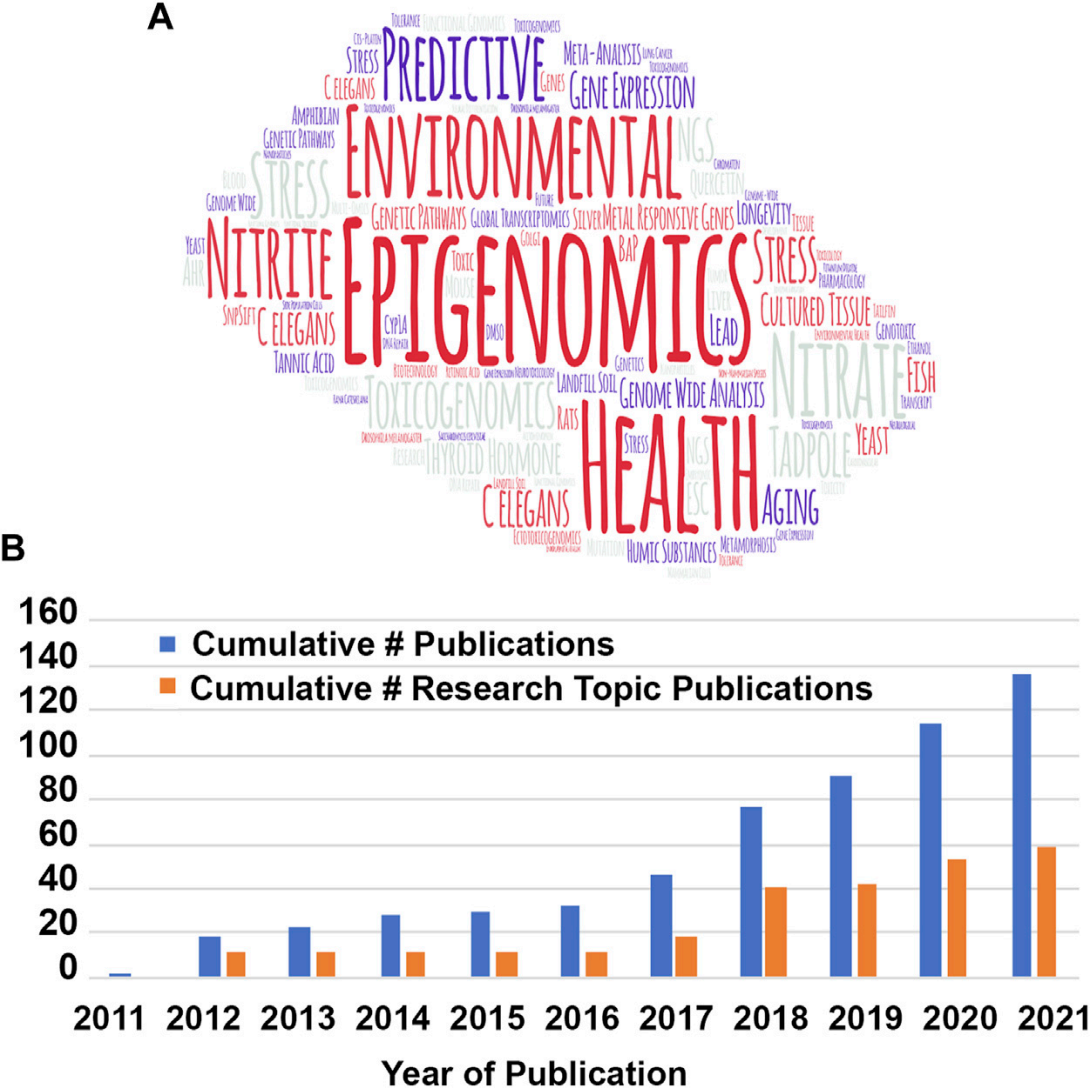
Cumulative number of publications and research topic publications. **(A)** Word cloud of titles from the first 25 of the 138 publications published in the first 10 years of Frontiers in Toxicogenomics/section. The size of the word is proportional to the frequency that word is used in the titles (figure made from http://wordart.com). **(B)**. Cumulative number of publications (all–blue; research topic–orange). Note that research topics currently include over half of the submitted publications.

This mini review is the first scientometric analysis of the Toxicogenomics and is part of a series of articles from Frontiers in Genetics Specialty Chief Editors celebrating the first decade of Frontiers in Genetics. To get started on this exercise, I decided that one of the best ways to find key words that represent this specialty journal is to make a so-called “word cloud” of all the words used in the titles of the first 25 articles, discarding common words like “and” and “the.” There are many varieties of word clouds, but most of them represent the words with highest frequency by the size of the word. To find the most representative key words for the initial articles of Toxicogenomis, I made a word cloud, using a freeware program called WordArt, of the first 25 journal article titles in Toxicogenomics that were published in the first 3 years of the journal (Figure 1A). In the word cloud, you see that the words “Epigenomics,” “Predictive,” “Environmental” and “Health” are the top words based on their frequency of usage. Smaller words such as “*C. elegans*,” “*Drosophila*,” “Rat,” “Zebrafish” and “Yeast” represent the model systems that were primarily used for toxicogenomics analyses in the initial 3 years of the journal. Some of the words are repeated in this version of the word cloud to fill in space, but the size of the word represents its frequency and not the number of repeats of the word. The articles published in the first 3 years are key to setting the theme of any journal, and that proved to be the case for Toxicogenomics because the word art strongly represents the key words in journal articles to this day.

If you look at the cumulative number of articles published, you see that the journal started out slowly in 2011 with only one article published, “Epigenomics in environmental health” by Drs. Christensen and Marsit, both of whom have served for several years as Associate Editors of Toxicogenomics (Christensen and Marsit, 2011). After 2016, when Frontiers in Genetics was listed in PubMed and Scopus citation indexes, the number of articles published increased dramatically from a total of 38 in the first 5 years to a total of 138 in 2021 (Figure 1B). We hope that Toxicogenomics continues this dramatic increase in growth. Of notice in the type of articles published in Frontiers in Genetics: Toxicogenomics section, in the first 5 years most of the articles were unsolicited submisssions, whereas in the past 5 years, over half of the publications were submitted as Research Topics. Frontiers in Genetics realized early on that this theme was true for all its specialty journals and has continued to push the publication of research topics and offers discounts to authors submitting to research topic solicitations.

**FIGURE 2:**
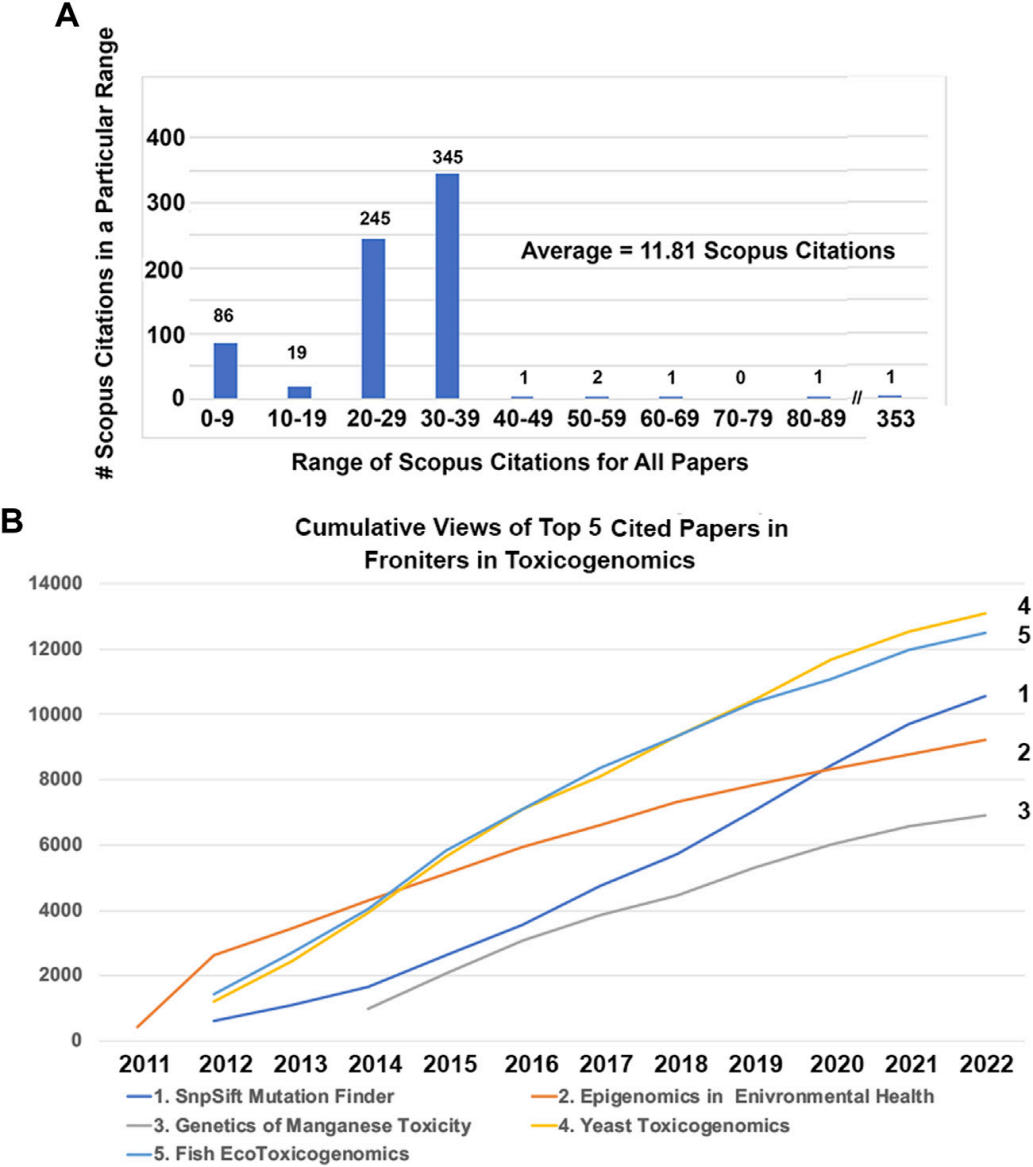
Scopus citations and views for top 5 cited publications in Frontiers in Toxicogenomics. **(A)** Number of Scopus citations for all publications in Frontiers in Toxicogenomics. The number of publications with 0–9, 10–19, et cetera Scopus citations are shown. The average number of Scopus citations is 11.81. One publication written by the author’s laboratory. **(B)** had 352 Scopus citations, which is an outlier compared with all of the other publications (Cingolani et al., 2012a). **(B)** Loop views of the publication with the largest number of Scopus citations. The publication “Using *Drosophila melanogaster* as a model for genotoxic chemical mutational studies with a new program, SnpSift” by the author’s laboratory had 353 Scopus citations and 8,951 views at the time of writing this review (Cingolani et al., 2012a). **(C)** Loop views of the publication with the second largest number of Scopus citations. The publication “Epigenomics in environmental health” by Drs. Christensen and Marsit had 86 Scopus citations and 8,464 views (Christensen and Marsit, 2011). **(D)** Loop views of the publication with the third largest number of Scopus citations. The publication “Genetic factors and mangenese induced neurotoxicity” by Dr. Michael Aschner’s laboratory, who was a co-Specialty Chief Editor for Frontiers in Toxicogenomics for 5 years, had 61 Scopus citations and 6,175 views (Chen et al., 2014). **(E)** Loop views of the publication with the fourth largest number of Scopus citations. The publication “Yeast toxicogenomics: genome-wide responses to chemical stresses with impact in environmental health, pharmacology, and biotechnology” by Dr. Sa-Correia’s laboratory had 59 Scopus citations and 11,994 views (Dos Santos et al., 2012). **(F)** The publication “Applications for next-generation sequencing in fish ecotoxicogenomics” by Dr. Denslow’s laboratory had 50 Scopus citations and 11, 296 views (Mehinto et al., 2012).

Next, I consider the top 5 articles published based on Scopus citations, which are listed for all 138 articles published from 2011 to 2021 in Supplementary Table S1. Of all 138 articles published in Toxicogenomics in the first 10 years, most of them had between 20 and 39 Scopus citations (Figure 2A). The top 5 articles based on Scopus citations were outliers with 353, 86, 61, 59, and 50 citations. The top article with an impressive 353 Scopus citations, 8,951 Loop views, and over 600 Google Scholar citations, was titled, “Using *Drosophila melanogaster* as a model for genotoxic chemical mutational studies with a new program, SnpSift” (Figure 2B) (Cingolani et al., 2012a). This program, SnpSift, was created by a talented bioinformatician in my laboratory, Dr. Pablo Cingolani, as a sub-program of his even-more cited program SnpEff, which has over 6,000 Google Scholar citations, making it one of the top 10 cited bioinformatics programs (Figure 2B) (Cingolani et al., 2012b).

A common theme among the top 5 cited publications in Toxicogenomics was that they were cited near the founding of the journal—2 were published in 2011, 2 in 2012, and one in 2014. This makes sense for the obvious reason that the longer a publication has existed, if it continues to be cited, it will accumulate more and more citations as the years pass. This is true for the second most cited publication, which was also the inaugural article in the journal, “Epigenomics in environmental health” by associate editors Drs. Christensen and Marsit, which had 86 Scopus citations and 8,464 views (Figure 2B) (Christensen and Marsit, 2011). The third most cited publication, which was published in 2012, “Genetic factors and mangenese induced neurotoxicity” by Dr. Aschner’s laboratory, who was a co-Specialty Chief Editor for Toxicogenomics section for 5 years, had 61 Scopus citations and 6,175 views (Figure 2B) (Chen et al., 2014). The fourth most cited publication, which was published in the slightly more recent year of 2014, “Yeast toxicogenomics: genome-wide responses to chemical stresses with impact in environmental health, pharmacology, and biotechnology” by Dr. Sa-Correia’s laboratory had 59 Scopus citations and 11,994 views (Figure 2B) (Dos Santos et al., 2012). This article was important because it encourage the submission of many more yeast and other model organism toxicogenomics articles in the subsequent years of the journal. The fifth most cited publication, “Applications for next-generation sequencing in fish ecotoxicogenomics” by Dr. Denslow’s laboratory had 50 Scopus citations and 11,296 views (Figure 2B) (Mehinto et al., 2012). This article was important in bringing ecology-related toxicology papers to the journal and was one of the first publications in the emerging field of ecotoxicogenomics.

### 2.2 Future perspectives

In the previous section we focused on Scopus citations which are biased to articles written in the early years of the journal. In order to better understand the future perspectives of the section, it is illustrative to discuss the recent 2018 publication, “Comparison of RNA-Seq and Microarray Gene Expression Platforms for the Toxicogenomic Evaluation of Liver From Short-Term Rat Toxicity Studies” by Dr. Liguori’s laboratory that had 39,906 views and 38,285 downloads so far (Supplementary Figure S1) (Rao et al., 2018). Dr. Liguori is a much-appreciated review editor of the journal. Analyses of the views (Supplementary Figure S1) and downloads (Supplementary Figure S1) of all the 138 articles published in Toxicogenomics specialty section shows that this article is a far outlier over all the other publications, several standard deviations above the mean.

Toxicology is a large field and Toxicogenomics covers only a limited subset of that field. Therefore, Frontiers Media decided in 2019 to start a new journal, Frontiers in Toxicology, to cover the whole field on Toxicology sciences.

The Editors of Frontiers Media, are in the midst of discussions to address bioinformatics issues, such as the need to harmonize big data and to require authors to use standardized vocabulary for their gene symbols (official gene symbols, Gene:ID, seqIDs) and chemical identifiers (such as MESH:ID, PubChem:ID, CAS:ID, catalog numbers from chemical companies) and cell lines. We also acknowledge that Frontiers Media Editors need to take an active role by implementing publication policies that ensure data FAIRness. The FAIR (findability, accessibility, interoperability, and reusability) principles emphasizes the capacity of computational systems to find, access, and reuse data with minimal human intervention because of the increase in volume, complexity, and speed of data creation. FAIRness is especially important in the fields of genomics and toxicology because of the logarithmic increases in data generation.

In summary, Toxicogenomics, while one of the smallest specialty section in Frontiers in Genetics, has grown tremendously over the past 10 years to one the top Toxicology journals. I am certain that the new specialty journal Frontiers in Toxicology will also soon emerge as another top toxicology journal for a more general audience. Toxicogenomics has paved a path for its success.

## Supplementary material

SUPPLEMENTARY FIGURE S1 The Future of Frontiers in Toxicology is Represented by a 2018 Paper on a Comparison of Gene Expression Methods for Rat Toxicity Studies. **(A)** Loop views of the publication with the largest number downloads and views. The publication “Comparison of RNA-Seq and Microarray Gene Expression Platforms for the Toxicogenomic Evaluation of Liver From Short-Term Rat Toxicity Studies” by Dr. Liguori’s laboratory had 39,906 views and 38,285 downloads so far from the time of publication in 2018 until November 2021 (Rao et al., 2018). **(A)** Graph of cumulative downloads for all publications in 2019 when Supplementary Table S1 was made. *, includes the abovementioned comparison paper from Dr. Liguori’s laboratory. **(B)** Graph of cumulative downloads for all publications until November 2021 when Supplementary Table S1 was made. **(C)** Graph of cumulative views for all publications until November 2021 when Supplementary Table S1 was made. **(B,C)***, indicates the abovementioned comparison paper from Dr.Liguori’s laboratory.
