# Erratum: 10 Years of Toxicogenomics section in Frontiers in Genetics: Past discoveries and Future Perspectives

**DOI:** 10.3389/fgene.2023.1213706

**Published:** 2023-05-30

**Authors:** Frontiers Production Office

**Affiliations:** Frontiers Media SA, Lausanne, Switzerland

**Keywords:** toxicology, toxicogenomics, epigenomics, environmental health, genomics, transcriptomics

Due to an error in the editorial process, this article was not proofread before publication. The article has now been proofread and the necessary journal-related corrections have been implemented. The previous version is available in the Supplementary Material of this article.

The article title has also been corrected.

The publisher apologizes for this mistake. The original version of this article has been updated.

