# Supplementary figures and images for: 10 Years of Toxicogenomics section in Frontiers in Genetics: Past discoveries and Future Perspectives
Source: Front Genet. 2022 Sep 12;13:979761. doi: 10.3389/fgene.2022.979761 (PMC9510767; doi:10.3389/fgene.2022.979761)

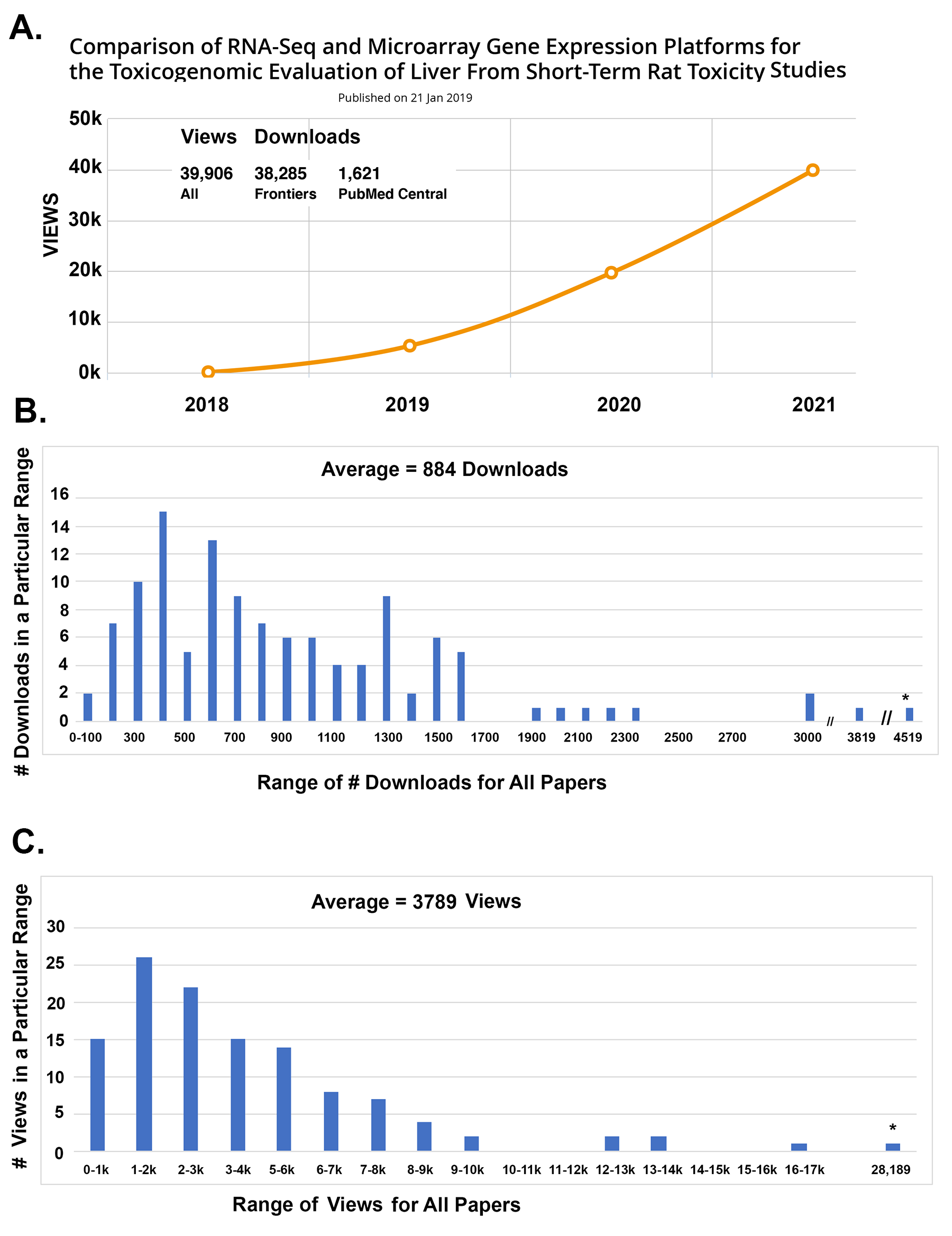

Supplement: Supplementary file 1 [file Image1.tif]
